# Role of Immune Cells in Biliary Repair

**DOI:** 10.3389/fimmu.2022.866040

**Published:** 2022-03-30

**Authors:** Tian Lan, Shuaijie Qian, Chengwei Tang, Jinhang Gao

**Affiliations:** ^1^ Lab of Gastroenterology and Hepatology, State Key Laboratory of Biotherapy, West China Hospital, Sichuan University, Chengdu, China; ^2^ Department of Gastroenterology, West China Hospital, Sichuan University, Chengdu, China

**Keywords:** cholangiopathy, biliary repair, liver progenitor cell, macrophage, hepatocyte-cholangiocyte transdifferentiation, immune cells

## Abstract

The biliary system is comprised of cholangiocytes and plays an important role in maintaining liver function. Under normal conditions, cholangiocytes remain in the stationary phase and maintain a very low turnover rate. However, the robust biliary repair is initiated in disease conditions, and different repair mechanisms can be activated depending on the pathological changes. During biliary disease, immune cells including monocytes, lymphocytes, neutrophils, and mast cells are recruited to the liver. The cellular interactions between cholangiocytes and these recruited immune cells as well as hepatic resident immune cells, including Kupffer cells, determine disease outcomes. However, the role of immune cells in the initiation, regulation, and suspension of biliary repair remains elusive. The cellular processes of cholangiocyte proliferation, progenitor cell differentiation, and hepatocyte-cholangiocyte transdifferentiation during biliary diseases are reviewed to manifest the underlying mechanism of biliary repair. Furthermore, the potential role of immune cells in crucial biliary repair mechanisms is highlighted. The mechanisms of biliary repair in immune-mediated cholangiopathies, inherited cholangiopathies, obstructive cholangiopathies, and cholangiocarcinoma are also summarized. Additionally, novel techniques that could clarify the underlying mechanisms of biliary repair are displayed. Collectively, this review aims to deepen the understanding of the mechanisms of biliary repair and contributes potential novel therapeutic methods for treating biliary diseases.

## 1 Introduction

**Figure 1:**
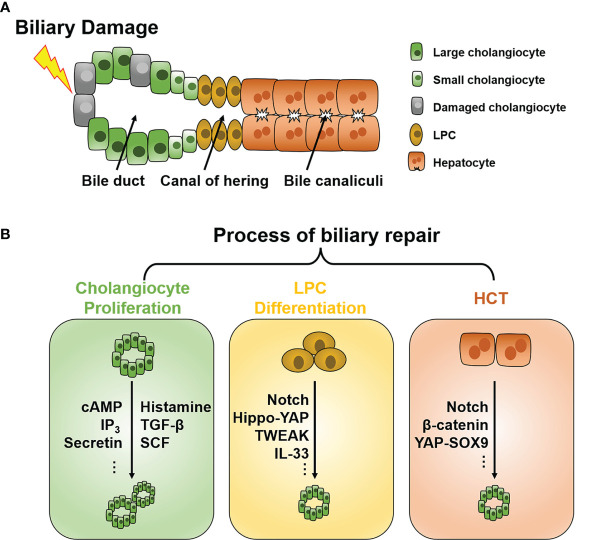
Schematic of the intrahepatic biliary tree structure and major mechanisms of biliary repair during biliary damage. **(A)** The intrahepatic biliary tree consists of bile canaliculi surrounded by apical membranes of hepatocytes, the canal of hering where LPC resides, and the bile duct lined by small and large cholangiocytes. **(B)** When subjected to insults, different repair mechanisms such as cholangiocyte proliferation, LPC differentiation, and HCT can be triggered depending on the pathological conditions. LPC, liver progenitor cell; HCT, hepatocyte-cholangiocyte transdifferentiation; cAMP, cyclic adenosine monophosphate; IP_3_, inositol 1,4,5-trisphosphate; TGF-β, tissue growth factor-β; TWEAK, TNF-like weak inducer of apoptosis; SCF, stem cell factor; YAP, Yes-associated protein; SOX9, Sry HMG box protein 9; IL, interleukin.

Cholangiocytes represent less than 5% of the cell population in the liver; however, they are indispensable for maintaining healthy liver function (1). Cholangiocytes line the biliary system from the canal of hering to the duodenum and are responsible for modifying bile formation and drainage (**Figure 1A**) (2). In addition, cholangiocytes secrete mucins, defensins, and immunoglobins during a quiescent state, protecting the biliary system from injury (3).

Genetic disorders and the majority of liver injuries could impair biliary function, namely cholangiopathies. Cholangiopathies are characterized by dysregulation of bile formation and bile flow (including bile acid circulation), biliary inflammation and fibrosis, and ultimately, cholangiocarcinoma (CCA) (4). When subjected to pathogenic factors, cholangiocytes can be activated and secrete various cytokines and chemokines, resulting in multiple pathophysiological changes and triggering cellular crosstalk, including activation of myofibroblasts and hepatic resident immune cells such as Kupffer cells, and infiltration of peripheral immune cells, such as monocytes, lymphocytes, mast cells, and neutrophils. These pathogenic changes in cholangiocytes eventually lead to biliary dysfunction and cholangiopathies (5). Cholangiopathies consist of a broad disease spectrum and can be classified etiologically as inherited, immune-mediated, infectious, vascular, malignant, and others. Although these diseases are relatively uncommon, they still cause considerable morbidity and mortality yearly. Even worse, limited therapies and treatments are available for cholangiopathies (4). Thus, research is warranted to elucidate the pathogenesis and discover promising therapeutic targets for cholangiopathies.

The liver possesses vigorous regenerative capability and is the only solid organ in the human body that can fully recover to a normal state under compensated injury (6). Liver regeneration is a complicated process regulated by multiple mechanisms, including classical signaling pathways as well as epigenetic and posttranscriptional modulation (6–8). As a vital part of the liver, the biliary system also harbors great reparative capacity. After biliary damage and consequent destruction of the biliary epithelium, a robust repopulation of cholangiocytes can be exerted to restore the equilibrium of the biliary epithelium. This specific tissue repair is defined as biliary repair.

Biliary repair involves diverse repopulating origins of cholangiocytes and complicated regulating systems and is essential for tissue homeostasis maintenance and disease regression. Nevertheless, aberrant and excessive biliary repair might play a role in carcinogenesis and the development of several cholangiopathies (9, 10). This review focused on the mechanisms and regulation of biliary repair in different cholangiopathies and highlighted the role of immune cell-mediated modulation of biliary repair. The role of aberrant biliary repair in the pathogenesis of some cholangiopathies is also summarized.

## 2 Mechanism of Biliary Repair

Under normal conditions, cholangiocytes are retained in the stationary phase and maintain a very low turnover rate to counterbalance occasional cell loss, such as apoptosis and senescence (11). When subjected to injuries, different repair mechanisms can be triggered depending on the pathological conditions. To date, three chief processes of biliary repair have been demonstrated (**Figure 1B**): 1) cholangiocyte proliferation, which is the replication of pre-existing cholangiocytes; 2) liver progenitor cell (LPC) differentiation toward cholangiocytes; and 3) hepatocytes with high plasticity transform to cholangiocytes, namely hepatocyte-cholangiocyte transdifferentiation (HCT). These processes generally orchestrate the reparation of biliary injury, although they contribute unequally in different cholangiopathies.

### 2.1 Cholangiocyte Proliferation

In a healthy liver, most cholangiocytes remain dormant mitotically, and proliferation occasionally occurs to achieve cellular equilibrium (11). In this review, we mainly focus on cholangiocyte proliferation in pathological conditions. The cell size of cholangiocytes gradually increases with morphological and functional changes along with the canal of hering to the large bile duct (12). This cell heterogeneity along with the biliary tree endows them with different responsiveness to insults (13). Major signaling pathways driving proliferation in both small and large cholangiocytes are Ca^2+^ and cyclic adenosine monophosphate (cAMP)-dependent (14). Secretin can bind to secretin receptors, and only large cholangiocytes express and elevate intracellular cAMP, initiating cell proliferation through the protein kinase A (PKA)/mitogen-activated protein kinase (MEK)/extracellular signal-regulated protein kinase1/2 (ERK1/2) signaling pathway (15–17). For small cholangiocytes, agents such as forskolin and follicle-stimulating hormone can also trigger cAMP increase and lead to cholangiocyte proliferation (17, 18). In addition, activation of the inositol 1,4,5-trisphosphate (IP_3_)/Ca^2+^ signaling pathway can also stimulate the proliferation of small cholangiocytes (14, 19).

Usually, large bile ducts are more susceptible to damage, resulting in compromised proliferative capability (20). Under this condition, small cholangiocytes can acquire the phenotype of large cholangiocytes and help to restore biliary epithelium as a compensatory method (21, 22). However, the mechanisms of such differentiation remain elusive. Heterogeneity of regenerating capacity was also found independent of cell size in cholangiocytes. It was indicated that cholangiocyte proliferation predominantly contributes to biliary repair. Proliferation is chiefly generated by a subgroup of cholangiocytes with persistent regenerating capability, which is not predestined but rather “stochastically” regulated during injury (23). In general, the biliary system does not uniformly proliferate as a whole due to significant cellular heterogeneity among the biliary tract, and this highly dynamic and adaptive replenishment process plays a fundamental role in biliary repair.

### 2.2 Liver Progenitor Cell Differentiation

Although cholangiocytes possess immense regenerative capacity, severe acute injury and prolonged chronic injury could exhaust their proliferative potential (24). The liver could also activate alternative reparation methods, such as the LPC expansion, to avoid abnormal repair, which eventually leads to ductular fibrosis and cirrhosis. LPCs, also known as oval cells, are considered bipotent progenitor cells that reside adjacent to the canal of hering and can differentiate into cholangiocytes or hepatocytes (25, 26). However, whether LPCs can give rise to cholangiocytes or hepatocytes under physiological conditions has obtained controversial results (27–30), so as in the biliary repair of cholangiopathies. While LPCs expand in response to insults, the cell fate of LPCs is chiefly determined by the type of liver injury (11, 31). When biliary damage causes regenerative capacity exhaustion of cholangiocytes, LPCs can differentiate toward cholangiocytes and impede hepatocyte differentiation simultaneously. Such lineage specification is modulated by an intricate signaling network, including the Notch (31), Wnt-β-catenin (31–34), and Hippo-Yes-associated protein (YAP) signaling pathways (35, 36).

LPC differentiation is also, to a large extent, modulated by their surrounding cellular milieu, called the “LPC niche”. The LPC niche comprises hepatic stellate cells (HSCs), macrophages and other immune cells, endothelial cells, and extracellular matrix (ECM) (37–39). Based on their adjacent distributions, HSCs (31, 40, 41) and macrophages (31, 42–44) can regulate parenchymal infiltration and differentiation orientation of LPC by intimate cellular crosstalk. In addition, ECM composition and remodeling during liver injury were also found to be crucial to LPC expansion and activation (45, 46). Though the contribution of LPCs for liver repair has been confirmed in several disease models, the cellular source of LPCs during liver repair is still elusive. LPCs originating from pre-existing hepatic stem cells is one of the hypotheses; however, compelling evidence, *i.e.*, exclusive molecular markers is lacking (47). Moreover, the contribution of LPCs to the regenerating biliary pool differs according to the liver injury model and lineage tracing methods utilized (**Table 1**).

**Table 1:** Summary of alternative biliary repopulating origins in biliary injury models.

Biliary repopulating origin	Species	Injury model	Methods of lineage tracing	Contribution to total biliary pool	Reference
LPC	Rat	BDL	transplanting DPPIV+ LPC into DPPIV- rat	Moderate contribution	(48)
	Mouse	DDC	SOX9-CreERT2-R26RYFP	major contribution	(49)
	Mouse	DDC, BDL	Foxl1-Cre; Rosa26-LacZ	30%-40% in BDL model	(50)
			β-gal, CK19 co-staining	~30% in DDC model	
	Mouse	DDC, BDL	SOX9IRES-CreERT2; Rosa26-LacZ mice	major contribution	(29)
	Mouse	DDC	OPN-iCreERT2; Rosa26RYFP	~36%	(51)
	Mouse	DDC	Foxl1-Cre; Rosa26-YFP	5%-15%	(52)
	Marmoset, mouse	DDC	Transplanting EGFP-positive marmoset LPCs into DDC treated Fah−/− mice	10%-20%	(53)
HCT	Mouse	DDC, DAPM	Mx1-Cre;Rosa26 mice injected with poly(I:C)	1.9% in DDC model	(54)
				4.7% in DAPM model	
	Mouse	DDC	CK19-Dre;Alb-CreER;IR1 mice	~5% in DDC model	(55)
	Mouse	DDC	Alb-CreERT2; Rosa26RYFP/YFP	62.6%-68.3%	(56)
	Mouse	DDC	Mx1-Cre;Rosa26 mice injected with poly(I:C)	~30%	(57)
	Mouse	DDC	A6 and HNF4α co-staining	~10%	(58)
	Mouse	DDC	transplanting Rosa26-mTmG hepatocytes into Fah-/- mice	8.7%–39.3% depending on donors	(59)
	Mouse	BDL, DDC	Alb-CreER; Rosa26-RFP	10.49% ± 0.59% in BDL model	(60)
				~11% in DDC model	
	Mouse	TAA	AAV8-TBG-Cre; Rosa26-YFP	16.16% ± 1.94%	(61)
	Mouse	BDL, DDC	AAV8-TBG-Cre; Rosa26-YFP	14.3%-48.1% in DDC model depending on the biliary markers	(27)
				2%-62.6% in BDL model depending on the biliary markers	
	Rat	BDL, DAPM	transplanting DPPIV+ hepatocytes into DPPIV- rat	1.75% in BDL model	(62)
				44.85% in DAPM+BDL model	
	Rat	BDL	transplanting DPPIV+ hepatocytes into DPPIV- rat	~30%	(63)
	Mouse	DDC	rAAV2/8-iCre; Rosa26-tdTomato	1.88%	(23)
	Mouse	BDL, DDC	AAV8-Ttr Cre; Rosa26-EYFP	None	(28)

HCT, hepatocyte-cholangiocyte transdifferentiation; LPC, liver progenitor cell; poly(I:C), polyinosinicepolycytidylic acid; DPPIV, dipeptidyl peptidase IV; DDC, 3,5-diethoxycarbonyl-1,4-dihydrocollidine; BDL, bile duct ligation; DAPM, methylene dianiline; TAA, thioacetamide; FACS, fluorescence-activated cell sorting; AAV, adeno-associated virus; β-gal, β-galactosidase.

### 2.3 Hepatocyte-Cholangiocyte Transdifferentiation

Another theory with nonnegligible evidence is that hepatocytes and cholangiocytes actually serve as facultative progenitor cells with reprogramming capacity and can transdifferentiate into each other. The intermediate phase of cells undergoing this process expresses both hepatocyte and cholangiocyte phenotypes and might be interpreted as LPCs, which seem inappropriate as they are not independent cell clusters and harbor considerable heterogeneity within the cluster (27, 47, 64). The more moderate theory is that hepatocytes or cholangiocytes first de-differentiate to LPCs and then differentiate to each other (65, 66). Each postulation obtains proponents and opponents, and in this review, we focus on those relevant to biliary repair.

Several studies failed to observe cell plasticity of lineage-tagged hepatocytes in the condition of biliary injury caused by bile duct ligation (28) or 3,5-diethoxycarbonyl-1,4-dihydrocollidine (DDC) diet model (23). Conversely, other researchers have confirmed HCT *in vivo* in rodent biliary injury models by utilizing the lineage-tracing technique. This HCT process seems to occur without LPC involvement. However, the proportion of HCT-derived cholangiocytes varied extensively in different models (**Table 1**). Transplanting labeled hepatocytes into the liver of recipients can also repopulate the biliary pool through HCT after biliary damage, whereas its contributions to the biliary pool remain varied (**Table 1**). During acute biliary injury, almost all hepatocytes are capable of HCT (67), while a subpopulation of Sry HMG box protein 9 (SOX9)-positive hepatocytes was identified as most potent in biliary reprogramming because they comprise 3% of all hepatocytes but contribute to nearly one-third of HCT (60, 68). These results correspond with clinical findings that cells coexpressing hepatocyte and cholangiocyte markers exist in liver samples from patients with cholangiopathies (69).

Although HCT derived cholangiocytes can acquire a genetic phenotype similar to that of *bona fide* cholangiocytes, it remains inconclusive whether these cells have sufficient biliary function. It was reported that cholangiocytes of HCT origin could obtain featured cellular polarity (positive staining of apical and basolateral markers of cholangiocytes, including primary cilia) (27) and even form mature bile ducts capable of bile drainage when the biliary system develops abnormally (70). In contrast, some studies found that HCT-derived cholangiocytes are functionally immature, and unable to coalesce with the pre-existing bile duct. These HCT-derived cholangiocytes might be a transient form that will revert to hepatocytes after injury subsides (23, 56, 59, 71). The possibility that these maladaptive cells contribute to CCA formation was also raised (9, 72).

In general, the role of HCT in biliary repair is not yet fully understood. It seems to be a double-edged sword that can respond to biliary damage and rescue the biliary system by replenishing the biliary pool. However, it might not be sufficient to form a mature bile duct to counterbalance compromised biliary proliferative capacity and function, even giving rise to carcinogenesis.

### 2.4 Other Sources of Biliary Repair

Except for the processes mentioned above, there are some less common sources of biliary repair. Some progenitor cells reside in the peribiliary gland in the extrahepatic biliary tree (73). They resemble LPCs in many ways, such as similar genetic phenotypes and are likewise capable of bipotent differentiation to hepatocytes and cholangiocytes. In comparison, they are primarily activated when large intrahepatic or extrahepatic bile ducts are affected (74–76). Moreover, it was reported that bone marrow stem cells can be recruited to the liver during several liver diseases and can differentiate into LPCs and cholangiocytes (77–79). Compared to the abovementioned process, these approaches may play a less important role in biliary repair. However, they remain worthy of further study as they, especially bone marrow stem cells, serve as a potential cellular therapy for liver disease (80).

## 3 Crosstalk Between the Immune System and Biliary Repair

Cholangiocytes are susceptible to various exogenous and endogenous insults and were once deemed innocent victims of liver injury. However, they are now unmasked as dynamic participants in the pathogenesis and development of several liver diseases (5). Cholangiocytes subjected to insults can switch from the quiescent form to the active form, which is also called “reactive ductular cells (RDCs)” and is characterized by an anomalous morphology and secretory phenotype (81). RDCs play a multifaceted role in orchestrating biliary repair by autocrine and paracrine signaling of several chemokines and cytokines (82). Similarly, immune cells have long been recognized as an essential player in tissue hemostasis and repair (83). Recent studies further expand our knowledge about the communication between cholangiocytes and the immune system in biliary repair.

In the quiescent stage, cholangiocytes contribute to immune function by secreting immunoglobin A and antimicrobial peptides into the bile duct (84). Under disease conditions, damage-associated molecular patterns originate from adjacent damaged hepatocytes or nonparenchymal cells, and pathogen-associated molecular patterns derived from the gut-liver axis and bloodstream can activate Toll-like receptors constitutively expressed on cholangiocytes and provoke far more vigorous biliary immune responses (85, 86). RDCs can release a repertoire of chemokines and cytokines, such as the CC-motif and CXC-motif chemokine family, interleukin (IL) -6, 8, 17, *etc.*, and can recruit and activate various immune cells such as T cells, macrophages, and neutrophils to the periductular niche (87–94). These immune cells in turn modify biliary repair by adjusting the proliferating capability of adjacent cholangiocytes (95), expansion and differentiation of LPC (31, 42–44, 96), or plasticity of hepatocytes (61), *etc.* Thus, the crosstalk between cholangiocytes and immune cells and the specific role of different immune cell subsets in biliary repair are summarized.

### 3.1 Macrophages

Macrophages are the most abundant immune cells in the liver and comprise liver-resident Kupffer cells located in the space of Disse and macrophages derived from monocytes recruited from peripheral circulation (97). Macrophages are functionally heterogenous and are traditionally categorized into the proinflammatory “M1” subtype and the anti-inflammatory “M2” subtype (98). However, accumulating evidence indicates that macrophages have a more complicated phenotype spectrum, as they perform manifold or even opposing functions and express both M1 and M2 markers concurrently during liver injury (99, 100). Macrophages generally play a dual role in the pathogenesis of cholangiopathies; for example, during cholestasis, macrophages can either be activated to release proinflammatory factors and promote liver fibrosis (101), or perform an enhanced anti-inflammatory response mediated by the bile acid signaling pathway (102, 103). The balance between the proinflammatory and anti-inflammatory effects of macrophages was shown to be associated with the severity of cholestasis diseases and played a crucial role in biliary repair (104).

In cholangiopathies, CC-chemokine receptor 2 (CCR2) positive macrophages can be recruited to the periductular niche by CC-chemokine ligand 2 (CCL2) and other chemokines released from injured cholangiocytes (90). These peribiliary macrophages can provoke cholangiocyte proliferation by inducing the expression of Integrin αvβ6 (ITGB6) on cholangiocytes (105). Additionally, macrophages can eliminate senescent cholangiocytes and cell debris by phagocytosis favoring cholangiocyte proliferation (106). In addition, surrounding macrophages can incite expansion of LPC by secreting TNF-like weak inducer of apoptosis (TWEAK) even in the absence of liver injury. Depletion of CD11b^+^ macrophages can ameliorate this effect (107). After LPC expansion, macrophages can further regulate the lineage specification of LPC by paracrine signaling (31).

Using single-cell RNA sequencing (scRNA-seq), functionally distinct subpopulations of macrophages were discovered, and the composition of the macrophage population changes dynamically during liver diseases (108). For example, TREM2^+^CD9^+^ profibrogenic macrophages expand in liver fibrosis and induce HSC proliferation and activation (109). MACRO^+^ and MACRO^-^ macrophages were also found responding differently to inflammatory stimulations (110). Immune-related genes expression of macrophages are significantly altered in the DDC mouse model, and macrophage interactions with other liver cells are largely inhibited (111). However, contributions of heterogenous macrophages to biliary repair during cholangiopathies are largely unknown at the single-cell level. Studies further dissecting the specific role of different subsets of macrophages in biliary repair are warranted.

### 3.2 Neutrophils

Neutrophils respond swiftly to liver injury and are the first immune cells recruited to the injury sites. Neutrophils are considered proinflammatory cells and are responsible for exacerbating tissue damage (112). The infiltration of neutrophils is ubiquitous in liver injury and is involved in many cholangiopathies (97). For example, neutrophils can directly interact with cholangiocytes *via* integrin β1 and elicit cholestasis (113). Neutrophils were also indicated as central immune cells in the pathogenesis of immune-mediated cholangiopathies such as primary biliary cholangitis (PBC) and primary sclerosing cholangitis (PSC) (114). Recently their contribution to tissue repair was also discovered. Neutrophils can clear the injury site by phagocytosis, degranulation, and neutrophil extracellular traps to create an appropriate milieu for tissue reconstruction (115). Moreover, monocytes can be recruited by neutrophil-released IL-37 and help tissue repair (116). Nevertheless, their specific role in biliary repair has scarcely been studied, and further research is needed.

### 3.3 Mast Cells

Mast cells, a kind of inflammatory cell capable of releasing histamine and other particles, are emerging as novel participants in cholangiopathies (117). The infiltration of mast cells was detected in close proximity to cholangiocytes during cholestasis, PSC, PBC, and CCA (118–120). *In vivo* studies using antagonists and gene knockout mouse models further revealed a mast cell-mediated histamine-tissue growth factor-β (TGF-β)/stem cell factor (SCF) axis in the pathogenesis of PSC and CCA (117, 121). Concerning biliary repair, mast cells mainly regulate cholangiocyte proliferation. Coculture of cholangiocytes with mast cells isolated from bile duct ligated (BDL) rats significantly increased cholangiocyte proliferation *via* mast cell-derived histamine (118). Moreover, *in vivo* H1/H2 histamine receptor blockage dampened the proliferation of small cholangiocytes and large cholangiocytes, respectively (119). Whether other mechanisms of mast cells also contribute to biliary repair remains unclear and needs more studies.

### 3.4 Lymphocytes

Lymphocytes encompass many cell types, including T lymphocytes, B lymphocytes, natural killer cells, *etc.* ScRNA-seq of the human liver revealed distinct transcriptomic profiles of intrahepatic lymphocytes compared to peripheral lymphocytes (109). In the condition of liver cirrhosis, phenotype shift was observed in T cells and natural killer cells, such as expansion of a SELL^+^CD4^+^ memory T cell population and loss of CD56^+^CD16^−^ natural killer cells population (109, 122). Moreover, significant T cell heterogeneity was observed in liver of CCA and PSC patients and associated with disease pathogenesis and prognosis (123, 124). In contrast, there was no significant phenotypic changes of intrahepatic B cells in cirrhotic livers (109).

Nevertheless, the effect of these lymphocytes on biliary repair remains largely unknown. Innate lymphoid cells (ILCs), a newly defined, less enriched lymphocyte population located on the mucosal surface, are involved in regulating cholangiocyte proliferation. IL-33 can trigger cholangiocyte proliferation in a mouse model of biliary atresia, and type 2 ILCs (ILC2s) and ILC2-secreted IL-13 were found downstream of IL-33 in this mitogenic effect, which was abolished in the absence of ILC2s (95). IL-13 was also reported to be potent to drive LPC expansion and differentiation toward biliary fate (125). ScRNA-sec further revealed that two CD45^+^ ILC2 subsets, canonical ILC2, and newly defined “biliary immature myeloid cells”, interaction played an important role in IL-33 induced cholangiocyte proliferation (126). However, 10 weeks of IL-33 injection resulted in biliary tumorigenesis in mice genetically predisposed to CCA (95). It seems that the effect of lymphocytes on biliary repair is less well-defined.

### 3.5 Hepatic Stellate Cells

HSCs, the primary producer of hepatic ECM, are also closely related to biliary repair during cholangiopathies (127). Therefore, we included HSCs and introduced their role in biliary repair. HSCs and infiltrated immune cells together constitute the LPC niche (39). Likewise, HSCs can be activated and transformed to collagen-depositing myofibroblasts through immune cells-mediated signaling pathways, such as those involving macrophage-derived TGF-β and platelet-derived growth factor (PDGF) (127). Myofibroblast activation and ECM deposition are essential for LPC expansion. Myofibroblast-secreted Jagged1 could promote LPC differentiation toward the biliary lineage in the condition of biliary injury (31, 41, 128). Moreover, myofibroblasts can also regulate HCT by interacting with laminin and ITGB6 (61). Except for the abovementioned functions, HSCs were even reported to gain progenitor features and function as bipotent LPCs to repopulate the liver parenchyma after partial hepatectomy (PHx) (129, 130). By utilizing scRNA-seq, functional zonation patterns and heterogeneous subpopulations of HSCs were discovered (131). However, its critical role in biliary repair remains to be elucidated.

**Figure 2:**
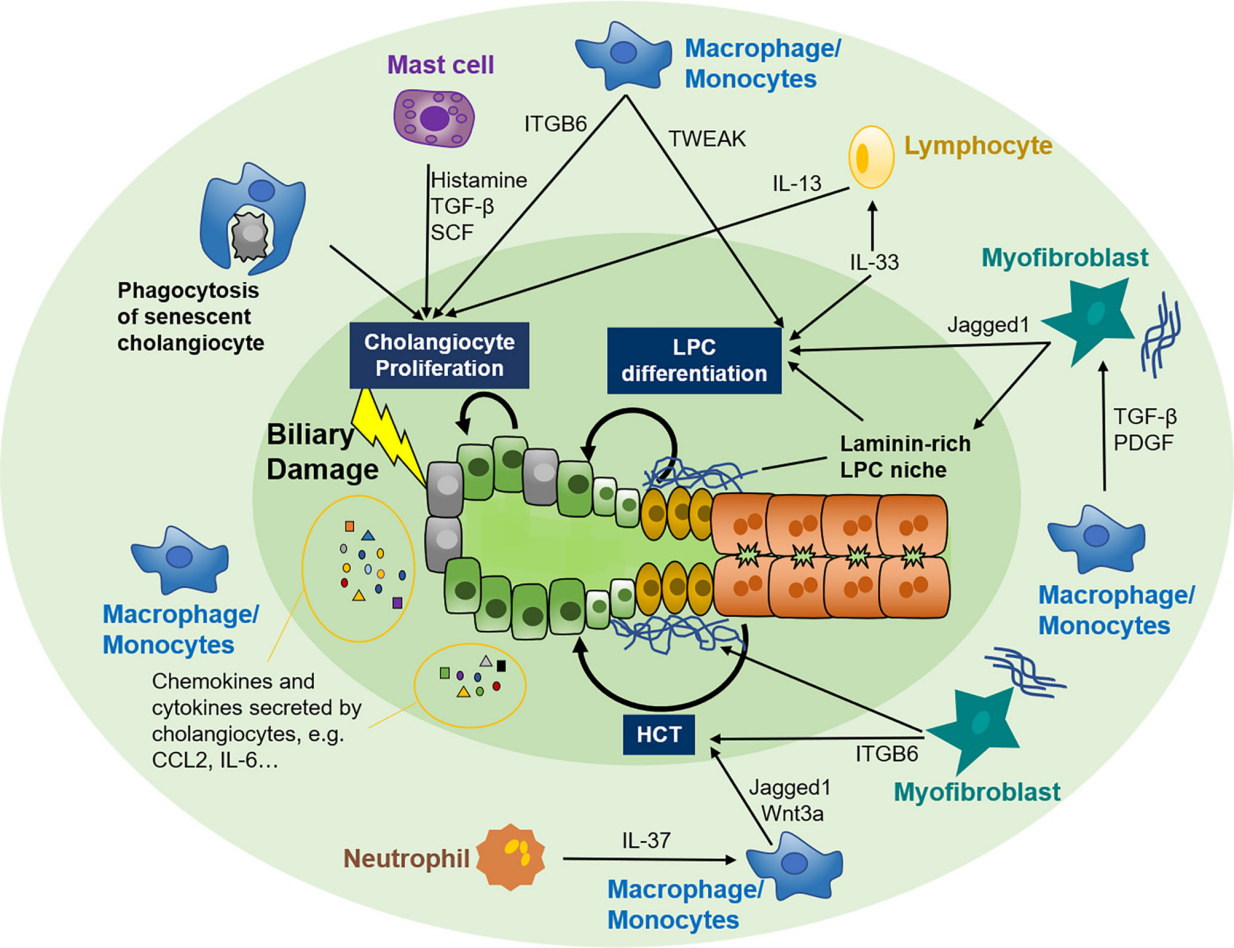
Crosstalk between the immune system and biliary repair. Cholangiocytes are susceptible to various exogenous and endogenous insults that facilitate the switch from quiescent to active. Active cholangiocytes can recruit various immune cells, including macrophages, mast cells, neutrophils, and lymphocytes, to biliary injury sites by autocrine and paracrine of several chemokines and cytokines. In turn, these immune cells modify biliary repair by adjusting the proliferating capability of adjacent cholangiocytes, expansion and differentiation of LPC, or plasticity of hepatocytes. Myofibroblasts are also involved in the crosstalk between immune cells and biliary repair. LPC, liver progenitor cell; HCT, hepatocyte-cholangiocyte transdifferentiation; TGF-β, tissue growth factor β; SCF, stem cell factor; IL, interleukin; TWEAK, TNF-like weak inducer of apoptosis; ITGB6, integrin αvβ6; CCL2, CC-chemokine ligand 2; CXCL2, CXC-chemokine ligand 2; PDGF, platelet-derived growth factor.

The role of different immune cells in biliary repair is summarized in **Figure 2**. Notably, immune cells are the key participants in the inflammatory response and have a double-sided effect on tissue repair. RDCs are highly proliferative to refurbish damaged biliary trees. Recruited immune cells could perpetuate the proliferation of RDCs, whereas protracted inflammation and excessive immune cell infiltration disturb biliary function and induce cell death (132, 133), ultimately leading to ductopenia caused by overbalanced apoptosis (134, 135). Similarly, cholangiocyte senescence, a mechanism to avoid neoplasia by irreversible cell cycle arrest, also has a secretory phenotype comparable to RDCs (5). Although senescence means decreased proliferation, mild senescence can provoke tissue repair by other mechanisms, including secretion of growth factors and proteins facilitating the expansion of LPCs and recruitment of immune cells regulating tissue repair (136, 137). Nonetheless, preserving senescence leads to tissue damage and impaired tissue repair (137). The immune cells show a double-edged sword role in biliary repair, and further studies that focus on the crosstalk of immune cells and cholangiocytes are urgently needed. As the heterogeneity of immune cells exists during biliary repair, it is also crucial to clarify the specific properties and factors secreted by these immune cells.

## 4 Role of Immune Cells in Biliary Repair in Cholangiopathies

Genetic disorders and liver injuries could impair biliary function, namely cholangiopathies. The role of immune cells in the development of cholangiopathies remains elusive. We next summarize the effect of biliary repair mediated by immune cells in cholangiopathies, such as immune-mediated cholangiopathies. As few studies are available on immune cells and biliary repair in inherited cholangiopathies, obstructive cholangiopathies, and CCA, other mechanisms of biliary repair are reviewed for these diseases.

### 4.1 Immune-Mediated Cholangiopathies

PBC and PSC are two major chronic cholestatic diseases caused by disordered immune conditions (82). Putatively, the pathogenesis of PSC is associated with the detrimental effect caused by the crosstalk of active cholangiocytes with innate and adaptive immune systems, which are activated by proinflammatory mediators originating from gut microbiota (138). Toxic bile acid was also proposed to contribute to PSC development (138). For PBC, a breach of biliary immune tolerance of the E2 subunit of the pyruvate dehydrogenase complex and an aberrantly activated immune response were postulated to play a central role in PBC pathogenesis (139). Despite these hypotheses, the pathogenesis of the two diseases remains incompletely understood, and effective treatment is lacking. Studies concerning biliary repair in these diseases are warranted to facilitate understanding of disease development and provide potential therapeutic targets.

#### 4.1.1 Cholangiocyte Activation

An increase in cholangiocyte activation and senescence along with the subsequent secretion of proinflammatory factors was observed in both PBC and PSC, indicating impaired biliary repair (140, 141). Overactivation of the Notch signaling pathway was found in both diseases, further indicating aberrant biliary repair conditions (142, 143). As immune-mediated diseases, the immune cells recruited by cholangiocyte secretion significantly altered the composition of intrahepatic immune cells and are involved in disease pathogenesis and regulation of biliary repair (144). ScRNA-seq of liver of PBC patients indicated that ORMDL3^+^ cholangiocytes displayed higher interaction with immune cells such as macrophages and monocytes, which might play a role in the pathogenesis of PBC (145).

#### 4.1.2 Macrophages

Macrophages induce ITGB6 expression on cholangiocytes in animal models of biliary epithelium injury and congenital hepatic fibrosis (105, 146). Furthermore, ITGB6 was critical to LPC function in animal models of sclerosing cholangitis mimicking PSC (147). The above results indicate a potential role of macrophages in the regulation of LPC-mediated biliary repair. Moreover, distinct LPC differentiation patterns were observed between PSC and PBC. Generally, LPCs exhibit a predominant biliary phenotype in PBC, while LPCs are more likely to differentiate into hepatocytes in PSC (148).

Moreover, expansion of progenitor cells in the peribiliary gland was also reported in PSC but not in PBC (149). This finding can be partly explained by the different distributions of biliary lesions. Interlobular bile ducts are mainly affected in PBC, thus LPCs residing in the canal of hering around the portal tract are activated to refurbish injured ductules. In contrast, large intrahepatic and extrahepatic bile ducts are the major damaged targets in PSC, favoring activation of peribiliary gland stem cells rather than LPCs (149). In addition, biliary stenosis and resultant cholestasis-induced hepatocytic injury in the PSC also drive LPCs toward hepatocyte differentiation (148). Another theory is associated with immune cell crosstalk with LPC. Macrophages can determine the lineage orientation of LPCs through secretion of the Wnt-β-catenin signaling pathway, which induces hepatocyte differentiation and antagonizes biliary differentiation by inhibiting the Notch signaling pathway (31). Correspondingly, the relative numbers of macrophages in PSC were found to be three-fold higher than those in PBC (150). Enriched Wnt3a positive staining was observed in PSC patients (148), indicating the intriguing role of macrophages against biliary repair in PSC.

Furthermore, macrophages might also contribute to disease regression by eliminating senescent cells (106). LPCs also contribute to disease regression by releasing extracellular vesicles containing microRNA (miRNA) lethal-7, which can inhibit cholangiocyte activation and biliary fibrosis in multidrug-resistant protein 2 (Mdr2) ^-/-^ mouse model resembling features of human PSC (151). For PBC, biliary differentiation of LPCs might be promoted by Notch signaling pathway activation through cellular crosstalk with myofibroblasts (31). A laminin-rich stem cell niche also contributes to LPC commitment to the biliary lineage in PBC (39). These observations indicate that macrophages play a crucial role in the development and biliary repair of PBC and PSC.

#### 4.1.3 Other Immune Cells and Epigenetic Factors

Other types of immune cells, such as mast cells recruited by RDCs, can prompt cholangiocyte senescence and proliferation, partly through histamine and TGF-β signaling pathways. However, mast cells might also lead to cholestatic liver injury in Mdr2 ^-/-^ mouse model (119, 121, 152). Another study found proinflammatory cytokines induced the upregulation of nitric oxide synthase 2 (NOS2) in cholangiocytes of PSC patients. NOS2 inhibits cAMP production and subsequent fluid secretion into the bile duct, thus exacerbating cholestasis. This might also impair cAMP dependent cholangiocyte proliferation (133). Whether other immune cells and inflammatory cytokines are also involved in the pathogenesis of PBC and PSC needs more solid evidence.

A recent study also indicated an important role of long non-coding RNAs (lncRNAs) in the pathogenesis of PSC. LncRNA ACTA-AS1 was found upregulated in cholangiocytes of PSC patients. ACTA-AS1 facilitated cholangiocyte proliferation by binding p300 and promoting transcription of proliferative genes through acetylates lysine 27 on histone 3 (153). Knockdown of ACTA-AS1 significantly impaired cholangiocyte proliferation, indicating a potential role of epigenetic treatment in cholangiopathies, although more validations are needed.

### 4.2 Biliary Repair in Inherited Cholangiopathies

Inherited cholangiopathies are a subset of cholangiopathies caused by hereditary or genetic factors, including Alagille syndrome (ALGS), polycystic liver diseases (PLD), biliary atresia (BA), *etc.* Inherited cholangiopathies are rare but can lead to chronic and progressive damage to cholangiocytes (10). The pathogenesis of inherited cholangiopathies remains elusive and effective treatment is limited. Thus, understanding the mechanism and regulation of biliary repair during inherited cholangiopathies is of great significance in expanding our knowledge of these diseases and searching for potential therapeutic targets.

#### 4.2.1 Alagille Syndrome

ALGS is an autosomal dominant dysmorphogenetic disorder caused by mutations in the Notch ligand Jagged1 or rarely in Notch receptor 2 (154). ALGS is a multisystem disorder involving the liver, vasculature, heart, eyes, and skeleton and is characterized by dysplasia of the intrahepatic bile ducts and cholestasis (155).

SOX9 is a biliary marker and regulator of biliary development (156). In ALGS patients, the SOX9 level was inversely correlated with disease severity (157). In a Jagged1*^+/–^* mouse model, the expression of SOX9 was decreased in the pericortical region of the embryonic liver (158). Removing one copy of SOX9 in Jagged1^+/–^ livers could enhance the ductular reaction, inflammatory cell infiltration, and fibrosis and decrease the number of cholangiocytes per portal vein (157). Additionally, SOX9 overexpression activates Notch2 expression, promotes bipotential mouse embryonic liver cell differentiation into cholangiocytes, and eventually improves the morphogenesis of biliary structures, while HCT was not influenced (157). Conversely, in the Alb-Cre^+/-^; Rbpj^f/f^; Hnf6^f/f^ mouse model, HCT contributed to the mature bile duct formation independent of the Notch signaling pathway. Additionally, TGF-β signaling pathway is another potent driver of the biliary system formation from hepatocytes (70). In the zebrafish model of ALGS, multipotent progenitors reside in the extrahepatic duct, contributing to intrahepatic duct regeneration by balancing the Jagged1/Notch and fibroblast growth factor signaling pathways (159).

Collectively, LPC differentiation plays a major role in ALGS and is mainly regulated by the Notch signaling pathway, while HCT also contributes to the development of ALGS. Many kinds of animal mutation models have been developed to exploit the role of the biliary system in ALGS. Organoid culture systems also improve the cell models from 2D to 3D structures to better explore the mechanism of liver diseases and repair (160). However, few studies have focused on the crosstalk of immune cells and surrounding cells in the development of ALGS.

#### 4.2.2 Polycystic Liver Diseases

PLD is an autosomal dominant polycystic disease caused by mutations in the PRKCSH or SEC63 genes, both of which are expressed in cholangiocytes (4). Additionally, cAMP is an essential regulator of dysregulated signaling pathways in PLD, which increases in cholangiocytes lining liver cysts. Takeda G protein-coupled receptor 5 (TGR5) is a bile acid receptor, which is expressed in sinusoidal cells, Kupffer cells, gallbladder epithelia, and cholangiocytes. TGR5 increases cAMP expression and promotes cell proliferation and cyst growth in PLD. Inhibition of TGR5 alleviates this phenotype (161). Additionally, depleting SOX9 leads to cholangiocyte hyperproliferation and induces the formation of hepatic cysts *via* the Wnt/β-catenin signaling pathway (162). Moreover, diminished intracellular Ca^2+^ level also leads to cholangiocyte hyperproliferation in PLD development. In contrast, this hyperproliferation could be inhibited by ursodeoxycholic acid, a kind of bile acid regulating the intracellular Ca^2+^ level through the phosphatidylinositol 3-kinase (PI3K)/protein kinase B (AKT)/MEK/ERK1/2 signaling pathway (163).

Inflammatory cytokines, such as IL-6 and IL-8, are also involved in the pathogenesis of PLD (164). In addition to traditional pathogenesis factors, epigenetic elements, such as miRNAs, a kind of small non-coding RNA oligonucleotides that can regulate cell survival and proliferation, also contributed to the development of PLD. About 80% of miRNAs were found reduced in PLD cholangiocytes, which resulted in the accumulation of miRNA-targeted proteins. MiR-345 targeted cell-proliferation proteins were found most significantly increased in PLD cholangiocytes, leading to cholangiocyte hyperproliferation and cyst growth (165). MiR-15a was also decreased in PLD cholangiocytes and was associated with cholangiocyte hyperproliferation and cyst growth by increasing Cdc25A expression (166).

In general, cholangiocyte hyperproliferation is the core mechanism in PLD. SOX9, cAMP, and Ca^2+^ play important roles in cystic growth *via* various signaling pathways. However, further studies that deepen the understanding of the physiological and pathological function of immune cells in PLD are still urgently needed.

#### 4.2.3 Biliary Atresia

BA is a leading cause of neonatal cholestasis and leads to advanced cirrhosis. BA is characterized by fibrosing obstruction of extrahepatic bile ducts, and its pathogenesis includes genetic factors, autoimmunity, abnormal fetal or prenatal circulation, inflammatory response, *etc.* (167). Moreover, epigenetic regulations, such as lncRNAs and miRNAs, are also involved in the development of BA.

H19 is a lncRNA and contributes to cell proliferation and differentiation. In BA patients, the expression of H19 in the liver and hepatic macrophages is significantly upregulated (168, 169). In addition, the expression level of hepatic H19 and serum exosomal H19 is positively correlated with the severity of liver fibrosis. In the BDL mouse model, knockout of H19 can inhibit cholangiocyte hyperproliferation and alleviate liver fibrosis *via* the sphingosine 1-phosphate receptor 2 (S1PR2)/sphingosine kinase 2 (SphK2) and lethal-7/high-mobility group AT-hook 2 (HMGA2) axes (168). H19 also regulates cholangiocyte proliferation *via* the Rho-GTPase signaling pathway by promoting the activation and polarization of macrophages (169).

The expression of miR-200s decreases in BA, while the expression of miR-124 increases in BA. MiR-200s promote the expression of IL-6 in cholangiocytes by inhibiting Forkhead Box A2 expression, while miR-124 reduces cholangiocyte proliferation *via* the inhibition of the IL-6/signal transducer and activator of transcription 3 (STAT3) signaling pathway (170). Thus, maintaining the balance of miR-200s and miR-124 may serve as potential targets for the treatment of BA. Inflammatory cytokines other than IL-6 are also involved in the development of BA. IL-33 is a member of the IL-1 cytokine family. In BA, increased IL-33 is beneficial to cholangiocyte proliferation *via* the IL-33/ILC2/IL-13 signaling pathway (95). In addition to the aforementioned epigenetic elements and inflammatory cytokines, transcription factors GATA6 can also control the proliferation and differentiation of cholangiocytes. Overexpression of GATA6 leads to HCT *in vitro* (171), and GATA6 deficiency suppresses cholangiocyte proliferation in BDL mice (172).

BA is a common cause of cirrhosis in children, and abnormal cholangiocyte proliferation is one of the most important mechanisms. Mechanically, lncRNAs, miRNAs, inflammatory cytokines, and transcription factors contribute to abnormal cholangiocyte proliferation. Apart from this, mitophagy, beta-amyloid deposition, and immune cells crosstalk are also involved in the development of BA (173–175).

### 4.3 Biliary Repair in Obstructive Cholangiopathies

Cholestasis induced by obstructive cholangiopathies is one of the important causes of chronic liver disease and cirrhosis. A significant role of LPC differentiation and HCT in biliary repair has been observed in animal cholestatic models.

#### 4.3.1 Hepatocyte-Cholangiocyte Transdifferentiation

In cholestasis models, activation of Notch-Hes1 signaling can promote HCT to form primitive ductules and induce the expression of SOX9 in mature hepatocytes (56). Furthermore, SOX9^+^ hepatocytes can differentiate into cholangiocytes with apical-basal polarity (positive staining of apical and basolateral markers of cholangiocytes) and form neo-lumens leading to biliary repair (27, 60). These HCT-derived cholangiocytes can transform back to hepatocytes after injuring (59). SOX9^+^ epithelial adhesion molecule (EpCAM)^−^ cells were found converted from mature hepatocytes with DDC injury. These cells were biphenotypic cells that can differentiate into functional hepatocytes and form bile duct-like luminal structures by converting into cholangiocyte-like cells and contributing to tissue repair (57).

The Wnt/β-catenin signaling pathway also regulates HCT-mediated biliary repair. DDC injury induces EpCAM^+^ cells to secrete Wnts, and activation of β-catenin with Wnt7a can promote HCT and reduce liver damage and mortality. Moreover, Kupffer cells and endothelial cells are necessary to activate β-catenin (58). However, some studies indicate that DDC only induces hepatocyte transdifferentiation to a biliary-like phenotype without EpCAM, cytokeratin 19 (CK19), and prominin-1 expression (23). One study even failed to identify hepatocyte-derived biliary epithelial cells in the left hepatic duct ligation mouse model (28). The reason may be that the damage induced by the left hepatic duct ligation mouse model instead of BDL is not severe enough to trigger HCT. Moreover, the study only detected cells without both CK19 and enhanced yellow fluorescent protein expression but did not attach importance to cholangiocyte-like cells.

PHx can stimulate hepatocyte proliferation and is a suitable model to study liver regeneration. In BDL and methylene diamiline (DAPM) after the PHx rat model, HCT was observed after injection of dipeptidyl-peptidase IV (DPPIV) positive hepatocytes into portal circulation (62). Another study showed that DPPIV positive hepatocytes could also be converted to cholangiocyte-like cells in the BDL rat model without PHx (63).

#### 4.3.2 Liver Progenitor Cell Differentiation

LPC differentiation into cholangiocytes also contributes to biliary repair in the BDL and DDC animal models. The winged-helix transcription factor Forkhead Box l1 (Foxl1) is a putative marker of LPC in mice. The expression of Foxl1 increases in the portal tracts with liver injury by BDL or DDC, and Foxl1 positive cells differentiate into hepatocytes and cholangiocytes (50). Deleting Foxl1 in LPC can decrease cholangiocyte proliferation, leading to more severe oxidative stress and liver function injury (52). In biliary repair, SOX9^+^ hepatocytes play an important role in HCT. Moreover, SOX9^+^ LPCs show biphenotypic potential to differentiate into cholangiocytes or hepatocytes (29). The ductular structures formed by LPC were observed degenerating after recovery from DDC injury (51). Biliary obstruction induced by BDL in rats led to exogenous DPPIV positive LPC differentiation into bile duct epithelial cells (48). Moreover, immortalized marmoset hepatic progenitor cells injected into DDC mice through the spleen were also capable of differentiating into cholangiocytes (53).

Cholestatic liver diseases are critical pathogenic factors resulting in cirrhosis and liver cancer, Whereas medicine management still faces huge challenges. Although accumulating mechanisms of HCT and LPC differentiation in cholestatic liver diseases have been discovered, new technologies are still needed to explore tissue microarchitecture in bile duct development and repair. Tissue engineering of the biliary tract can recapitulate site-specific characteristics of cholestatic liver disease and allow the study of the interrelation of bile ducts with other systems and cell-to-matrix interactions (176).

### 4.4 Biliary Repair in Cholangiocarcinoma

CCA is a biliary epithelial tumor categorized according to anatomical location as intrahepatic cholangiocarcinoma (iCCA) and extrahepatic cholangiocarcinoma (ECC). CCA is the second most frequent type of hepatic malignancy after hepatocellular carcinoma (HCC), accounting for 10% to 20% of newly diagnosed primary liver cancer cases (177). Abnormal biliary repair and chronic inflammation are known to be involved in the development of CCA.

#### 4.4.1 Intrahepatic Cholangiocarcinoma

iCCA is located proximally to the second-order bile ducts within the liver parenchyma, originating from mature cholangiocytes and hepatocytes (178). Abnormal cholangiocyte proliferation is the primary source of tumor cells in iCCA. IL-6 is a pleiotropic inflammatory cytokine and plays an important role in tumorigenesis and cancer progression. IL-6 is highly expressed in human iCCA (179) and contributes to CCA cell proliferation and invasion through the IL-6/STAT3 signaling pathway (180, 181). Furthermore, loss of c-Jun N-terminal kinases (JNKs) in hepatocytes affects cholesterol metabolism and bile acid synthesis, conjugation, and transportation (182), leading to overexpression of IL-6. Deletion of JNK1 and JNK2 contributes to diminished HCC and promotes iCCA development by stimulating cholangiocyte hyperproliferation (182, 183). It seems that the JNK-STAT-IL-6 signaling pathway is crucial for cholangiocyte proliferation in the development of iCCA.

Moreover, LPC proliferation is also involved in the development of iCCA. Brahma-related gene 1 (BRG1) encodes the enzymatic subunit of the Switch/Sucrose Non-Fermentable complex and contributes to stem cell maintenance and tumor development. BRG1 expression is upregulated in iCCA and enhances the Wnt/β-catenin signaling pathway to promote LPC proliferation, while inhibiting BRG1 prevents iCCA development (184). Furthermore, LPC regulated by macrophages is also involved in biliary repair in immune-mediated cholangiopathies and may be associated with CCA. Expression of TWEAK increases with the maturation of macrophages and promotes LPC proliferation and ductular reactions (107). In CCA, the TWEAK/Fibroblast growth factor-inducible molecule 14 signaling pathway provokes the recruitment and polarization of macrophages by increasing monocyte chemoattractant protein 1.

HCT also contributes to the development of iCCA. Notch2 is the major determinant of transdifferentiation of hepatocytes into malignant cholangiocytes in mice (185). In the thioacetamide (TAA) mouse model, Kupffer cells can express Jagged1 transiently to activate Notch signaling and induce HCT leading to the development of iCCA (186). Tumor necrosis factor receptor-related factor 3 (TRAF3) is a highly versatile regulator of organ development and tissue homeostasis *via* the tumor necrosis factor receptor family, the IL-1 receptor family, and the retinoic acid-inducible gene I -like receptors family in immune responses (187). In iCCA patients, low TRAF3 expression is associated with a poor prognosis. In liver-specific *Traf3*- and *Pten*-deficient mice, inactivation of TRAF3 induces HCT to promote iCCA development *via* NF-κB inducing kinase (NIK) upregulation, and NIK inhibition suppresses cholangiocyte overgrowth (188).

iCCA accounts for approximately 20% of all CCAs (189). Cholangiocyte proliferation, LPC differentiation, and HCT are all involved in the development of iCCA. However, mechanism studies are warranted to discover effective therapeutic targets in favor of the survival of iCCA patients.

#### 4.4.2 Extrahepatic Cholangiocarcinoma

ECC is divided into perihilar cholangiocarcinoma (pCCA) and distal cholangiocarcinoma (dCCA). pCCA is localized between the second-order bile ducts and the insertion of the cystic duct into the common bile duct, while dCCA is confined to the common bile duct below the cystic duct insertion (190). Several studies have focused on inflammatory cytokines in the carcinogenesis of ECC. IL-33 is a member of the IL-1 cytokine family and plays a crucial role in innate and adaptive immune responses, inflammatory responses, dysmetabolism, cardiovascular disorders, and cancer (191). In the liver, IL-33 promotes epithelial repair by the proliferation of hilar bile ducts *via* activation of the IL-33/ILC2/IL-13 axis (95). Consistently, IL-33 induces cholangiocyte proliferation from extrahepatic bile ducts but not intrahepatic bile ducts in human cholangiocyte cell lines (95). Additionally, IL-33 promotes ECC formation in mice with mutations of Kras and TGF-β Receptor 2 *via* activation of AKT and YAP, which predispose them to CCA (95, 192). IL-33 also upregulates the expression of IL-6, further accelerating the formation of CCA with activation of AKT and YAP (193). It seems that IL-33 could promote ECC formation by coordinating with other proinflammatory signaling pathways. ECC accounts for approximately 80% of all CCAs with poor prognoses (189). Studies in ECC are currently focused on clinical management and therapeutic targets. Further mechanisms studies on biliary repair and immune cell crosstalk are also needed.

## 5 Potential Novel Techniques in Biliary Repair

Although accumulating studies have proven dynamic biliary repair during cholangiopathies, the contributions of different regenerating pools of cholangiocytes remain an open question. Related studies are summarized in **Table 1**. More novel techniques remain needed to clarify the underlying mechanisms of biliary repair.

### 5.1 Lineage Tracing Techniques

In many studies, conclusions have been drawn utilizing the lineage tracing technique by labeling specific cell subsets, which enables researchers to distinguish the hepatocyte or biliary origin of regenerating cholangiocytes. The lineage tracing technique is a powerful tool to trace the progeny of certain cell types and reveal cell plasticity. However, one common drawback exists: the lack of exclusive cell biomarkers. For example, the most frequently adopted biomarkers of LPC, such as SOX9, EpCAM, and osteopontin, are shared by cholangiocytes or other liver cell types. Therefore, they are insufficient to detect LPC identity and might bias the conclusions, particularly in research regarding biliary repair (38). The identification of more specific and sensitive makers will benefit the understanding of liver regeneration. A feasible resolution is the application of a dual-recombinase system. Combining two separate recombinase systems, Dre-rox and Cre-lox, could effectively avoid non-specific labeling in lineage tracing (194). One study intercrossed CK19-Dre mice with Alb-CreER mice and yielded CK19-Dre/Alb-CreER/IR1 mice which showed significantly increased labeling accuracy of hepatocytes and cholangiocytes and further confirmed the appearance of HCT in DDC and BDL models (55). Another study concerning biliary repair designed a different labeling strategy using a dual-recombinase system. They labeled SOX9^+^ hepatocytes by SOX9-CreER; HNF4a-DreER mice and found that hepatocytes highly expressing SOX9 were the most vigorous origin of HCT during BDL and DDC induced biliary injury (60).

### 5.2 Single-Cell Related Techniques

Due to varied biliary injuries, pathological changes, and profound cell heterogeneity, cholangiocytes respond diversely to mitogenic mediators, and different repair processes are present in cholangiopathies. Additionally, LPC, hepatocytes, and liver resident or infiltrated immune cells also present varied subtypes and contribute differentially to biliary repair. However, traditional experimental tools such as immunostaining or RNA-sequencing are insufficient to reveal cell heterogeneity. In this case, scRNA-seq is a powerful technique to unmask transcriptional differences at the single-cell level. Utilizing scRNA-seq, researchers further confirmed heterogeneity within cholangiocytes and hepatocytes during cholangiocyte proliferation and HCT induced by biliary injury. It is identified that YAP enriched in a subset of liver epithelial cells dynamically drives liver regeneration (195). It is also suggested that some broadly-used biliary markers such as CK19 were inconsistently even low expressed in cholangiocytes. This might account for the contradictory results of studies applying these markers as biliary labels in lineage tracing (**Table 1**). Hepatic immune cells, as crucial regulators of biliary repair, also present significant heterogeneity (196, 197). scRNA-seq and more advanced techniques such as single-cell lineage tracing combined with single-cell transcriptomics (198), single-cell chromosome immunoprecipitation-sequencing, and single-cell assays for transposase-accessible chromatin sequencing are waiting for further application to dissect the role of different subsets of cells involved in biliary repair and their specific regulatory mechanisms.

### 5.3 Liver Organoids

The liver organoid model might also serve as a novel therapeutic method and experimental tool for liver injury and repair. Organoids are typically defined as *in vitro* three-dimensional organ-like architectures derived from isolated stem/progenitor cells and in many aspects superior to traditional two-dimensional culture systems (199). Liver organoids were reported to contain hepatocytes, cholangiocytes and bile ducts of normal function, which enable better recapitulation of liver disease and liver regeneration (200). Ductal organoids and hepatocyte organoids could be generated using a culture medium supplemented with different growth factors and could help to enhance the understanding of cholangiocyte proliferation and HCT (201). Moreover, liver organoids are currently deemed potential candidates for transplantation therapy for liver disease. Transplantation of ductal cell-derived and primary hepatocyte-derived liver organoids yielded promising results as they perform considerable repopulation capacity of liver parenchyma, including biliary tree, and physiological functions, such as albumin secretion (202–205). Notwithstanding, the possibility that transplantation therapy exacerbates liver disease by activating the LPC niche and raising cancer risk has not been fully ruled out (206). Thus, there is still a long way to go before the clinical application of cell transplantation therapy for liver diseases.

## 6 Summary and Prospects

The role of immune cells in the regulation of biliary repair is highlighted in this review. As mentioned above, immune cells, such as Kupffer cells, monocytes, lymphocytes, neutrophils, and mast cells, modulate cholangiocyte proliferation, LPC expansion, and differentiation as well as HCT. What is thought-provoking is that unbalanced biliary repair might escalate liver injury and harbor the risk of carcinogenesis (38, 207). Ductal-mesenchymal interplay was reported as a potential rheostat to control the extent of biliary repair and equilibrate its contradictory effects (208). However, the underlying signaling pathways and other candidate mechanisms that decide the timing of initiation and suspension of biliary repair are largely unknown and worth further study.

Following the discovery of the considerable regenerating capability possessed by LPC, hepatocytes, and cholangiocytes, transplanting these cells into the injured liver in aid of compromised regeneration seems a feasible and tempting treatment strategy. However, the results of hepatocyte transplantation were unsatisfactory as the regenerating effect was limited with fluctuating duration (80). In contrast, transplantation of LPCs that could differentiate into functional liver parenchymal cells yielded more promising results in the acute liver injury model (204). More studies are still needed to evaluate the potential clinical use of cell transplantation in the treatment of cholangiopathies.

Currently, several animal models of biliary injury have been established and extensively help investigation of the mechanisms of biliary repair and potential treatments. Unfortunately, they share some drawbacks, such as nonconformity in pathological and immunity changes with human patients (209). Thus, animal models better recapitulating the pathophysiological conditions of clinical patients would greatly benefit further unveiling the detailed machinery of biliary repair.

Although an increasing number of studies have shed light on our comprehensive understanding of the biliary repair, several vital questions remain to be solved: 1) Are LPC and HCT-derived cholangiocytes functionally competitive? 2) What mechanism determines the proportion of newly regenerated cholangiocytes from different origins? 3) During HCT, does the hepatocyte directly transform into a cholangiocyte or first undergo an intermediate progenitor-cell-like phase? 4) To what extent do immune cells regulate biliary repair? What is the underlying mechanism? 5) What mechanism suspends biliary repair and avoids excessive repair-induced carcinogenesis? Thus, what we already know about biliary repair is still far from the truth. Further studies are still urgently needed to fully understand the underlying mechanism and translate these observations into the clinical treatment of cholangiopathies.

In conclusion, this review summarizes the main regeneration methods of biliary repair and their varied regulations in different cholangiopathies. Great efforts are still needed to expand our knowledge regarding the detailed mechanisms of biliary repair in a broad spectrum of cholangiopathies and exploit promising therapeutic targets.

## Author Contributions

JG and CT conceived and supervised the study; TL, SQ and JG analyzed articles and finalized the figures.TL, SQ, CT and JG wrote the manuscript. All authors contributed to the article and approved the submitted version.

## Funding

This work was supported by the National Natural Science Fund of China (82170623, 82170625, U1702281, 81873584, 82000613, and 82000574), the National Key R&D Program of China (2017YFA0205404), Sichuan Science and Technology Program (2020YJ0084 and 2021YFS0147), and the 135 projects for disciplines of excellence of West China Hospital, Sichuan University (ZYGD18004).

## Conflict of Interest

The authors declare that the research was conducted in the absence of any commercial or financial relationships that could be construed as a potential conflict of interest.

## Publisher’s Note

All claims expressed in this article are solely those of the authors and do not necessarily represent those of their affiliated organizations, or those of the publisher, the editors and the reviewers. Any product that may be evaluated in this article, or claim that may be made by its manufacturer, is not guaranteed or endorsed by the publisher.
